# A Single Teaching Hospital Experience With Absorbable LATERA Implant for Nasal Valve Collapse

**DOI:** 10.7759/cureus.106376

**Published:** 2026-04-03

**Authors:** Mohammed Salem, Khaled Elgogary, Jiak-Ying Tan, Abdelrahman Ezzat Ibrahim

**Affiliations:** 1 Otolaryngology, United Lincolnshire Hospital NHS Trust, Lincoln County Hospital, Lincoln, GBR

**Keywords:** absorbable nasal implant, functional rhinoplasty, latera implant, nasal obstruction, nasal valve collapse

## Abstract

Introduction: Nasal valve collapse (NVC) is a common cause of nasal obstruction that significantly impairs quality of life (QoL), which is traditionally treated via functional rhinoplasty. There is, however, no consensus on the best management for NVC. This paper describes the experience of a single district centre using the LATERA implant (Stryker, Kalamazoo, MI, USA) to treat NVC by evaluating its efficacy and safety. Complications and patient satisfaction were evaluated as secondary outcomes.

Methods: A prospective study was conducted between 2022 and 2025. Eligible adults were recruited to undergo LATERA implant placement. The primary outcome of efficacy and safety of the LATERA implant was evaluated via the change in the Functional Rhinoplasty Outcome Inventory-17 (FROI-17) scores from baseline to six months post-procedure.

Results: A significant improvement in FROI-17 scores was observed in 90.9% (n=20) of patients, with a mean score decrease of 38.5 points (p<0.01). No major complications, such as infection or implant extrusion, were recorded.

Conclusion: The LATERA implant is a safe and effective intervention for NVC, showing a high success rate in significantly improving procedure-specific QoL in our single-centre study. These findings support its role as a valuable minimally invasive option, although further detailed comparative studies are warranted.

## Introduction

Nasal obstruction is one of the most common presentations referred to otolaryngologists. This can be caused by reduced airflow secondary to resistance in the nasal cavity. There are many structures within the nasal cavity that can potentially cause obstruction, including nasal valve collapse (NVC). Nasal valves are subdivided into external and internal valves. The internal valve is known as the 'isthmus nasi', which is an elongated 10 to 15-degree narrow opening between the caudal portion of the superior lateral cartilage and septum. This is also the area with the highest airflow resistance. The external valve is formed by the alar cartilage, connected tissues, and the columella medially at the nostril. Its movement regulates the airflow within the nasal vestibule [1,2]. There are two types of NVC: static and dynamic disorder. Aetiologies of static disorder are caused by obstruction from underlying structures which do not change during breathing, including hypertrophy of the head of the inferior turbinate, nasal septal deviation, etc. In dynamic disorder, the nostrils collapse inwards during inhalation secondary to weak cartilage or tissue [2].

Patients with NVC experience chronic nasal obstruction refractory to medical management such as topical corticosteroids or antihistamines. This affects patients' quality of life, sleep quality, and daily activities. Hence, functional rhinoplasty with cartilage grafting (e.g., spreader or alar batten grafts) remains the traditional surgical standard, although currently, there is no consensus on the best approach to treat NVC [3]. This is mainly due to variability in surgeons’ preferences, patients’ anatomy, and disease severity. Functional rhinoplasty comprises a variety of invasive procedures targeting different nasal deformities, which typically require general anaesthesia and prolonged recovery and can potentially impact patients' aesthetic appearance [4]. Furthermore, traditional grafting is associated with notable revision rates, reported at 5-10%, often due to graft displacement or resorption [5]. To provide a minimally invasive alternative, the LATERA implant (Stryker, Kalamazoo, MI, USA), a bioabsorbable implant designed to tackle NVC, was developed in 2016 to treat NVC [6].

Although LATERA was introduced in 2016, it remains a relatively new procedure in the United Kingdom (UK), especially in district hospitals. This study aims to describe the experience of a district hospital in the UK in using the LATERA implant to manage NVC. We evaluate its efficacy and safety in patients with isolated NVC. We will also discuss its distinct advantages over traditional grafting techniques, particularly concerning resource efficiency and alignment with NHS priorities for outpatient care.

## Materials and methods

Study design

**Figure 1:**
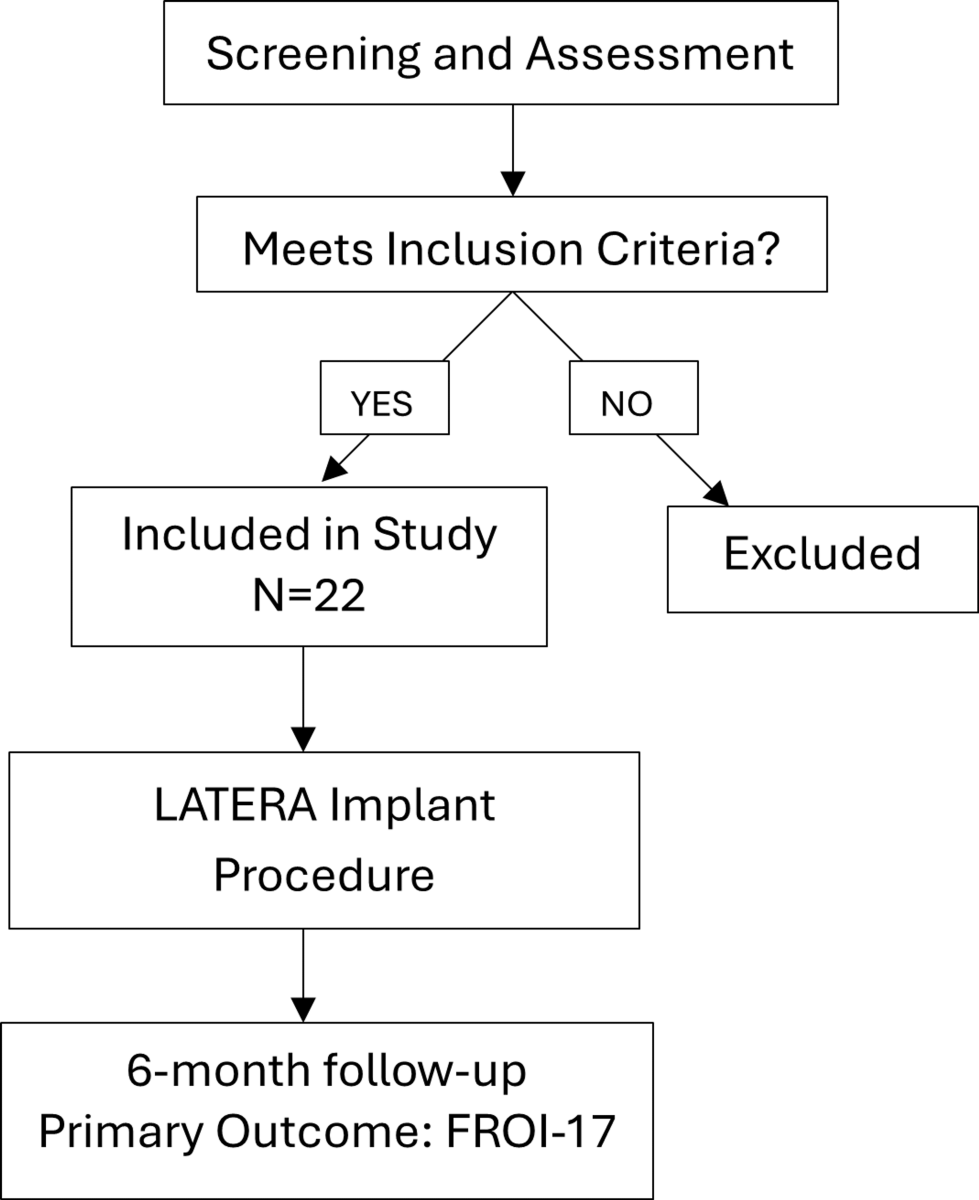
Study Process Flow Chart FROI-17: Functional Rhinoplasty Outcome Inventory-17

A prospective, single-centre study was conducted at the United Lincolnshire Teaching NHS hospitals between January 2022 and January 2025. Adult patients (>18 years) who were diagnosed with lateral wall NVC, unresponsive to at least eight weeks of medical therapy (topical steroids), were identified. Exclusion criteria included significant allergic rhinitis not controlled by medication, previous nasal valve surgery, and pregnant patients (Figure 1).

Outcome measures

The Functional Rhinoplasty Outcome Inventory-17 (FROI-17) was selected to assess multidimensional impacts over the Sino-Nasal Outcome Test (SNOT-22) for our study. FROI-17 is more structural nasal airflow-specific, whereas SNOT-22 is a questionnaire that is more sinusitis disease-specific. FROI-17 comprises 17 items across functional and psychosocial domains, including nasal obstruction, sleep disturbance, social interaction, and emotional well-being. Scores range from 0 to 100, with lower scores indicating a better quality of life. The primary outcome was the change in the FROI-17 score from baseline to six months post-procedure. A minimal clinically important difference (MCID) for the FROI-17 is considered a change of 15 points [7]. The FROI-17 is an open-access instrument, freely available for academic research and clinical use without licensing requirements, as specified by the original authors [7]. Its validity, reliability, and responsiveness have been confirmed in prospective studies, establishing it as a valuable tool for outcome measurement in septorhinoplasty populations [8].

Procedure-specific complications were recorded. Patient global satisfaction was recorded on a five-point Likert scale (Very Satisfied, Satisfied, Neutral, Dissatisfied, or Very Dissatisfied) at six months post-procedure.

Statistical analysis

Data were then analysed using IBM SPSS Statistics for Windows, Version 28 (Released 2021; IBM Corp., Armonk, New York). Pre- and post-operative FROI-17 scores were analysed using a paired t-test. A p-value of <0.05 was considered statistically significant. Descriptive statistics were used for demographic data and complication rates.

Surgical steps

Pre-procedure Preparation

Patients are positioned either in a seated position if under local anaesthesia (LA) or in a supine position if under general anaesthesia (GA). If the procedure is done under LA, Lignospan is injected inside the nose to anaesthetise the nasal wall completely. The implant path is marked and identified. An implant positioning guide is used to mark the target location for the implant's anchor (cauda edge of the maxilla bone) and tip (fibrofatty tissue near the lower lateral cartilage).

Device Setup

The LATERA implant (a 24 mm long, 1 mm diameter rod with a forked end) is loaded into a 16-gauge hollow delivery cannula. This collapses the forked end for insertion.

The tip of the delivery cannula is inserted through the nostril and into the lateral nasal wall submucosal plane with the pivot orientation aligned to rest on the nasal bone periosteum, following the pre-marked trajectory. The goal is to position the cannula tip, allowing the implant's forked end to anchor near the transition between the maxilla bone and cartilage.

Implant Deployment

The deployment plunger on the device handle is depressed to release the implant from the cannula. As the cannula is withdrawn, the remaining cylindrical implant is laid down along the path to support the upper and lower lateral cartilages. This process is repeated on the other side if bilateral treatment is required.

Closure/Dressing

No suture is required for closure. Paper-tape strips may be placed over the nose for support for approximately a week.

## Results

**Figure 2:**
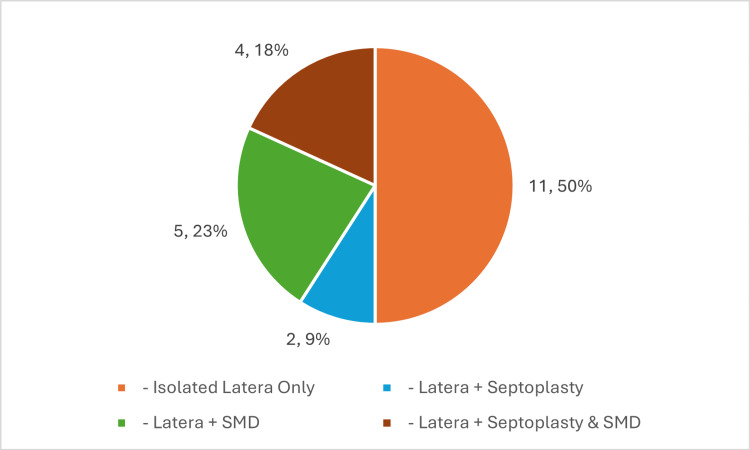
Distribution of Isolated Procedures and Concurrent Procedures

This study included a total of 22 patients. The mean age was 44 years (range 29-61) with equal distribution between male and female patients; 50% of patients had an isolated LATERA implant, with the remaining 50% undergoing concurrent procedures, e.g., septoplasty, submucosal diathermy, or both, under the same anaesthesia (Figure 2). 68% of procedures were carried out under GA.

**Figure 3:**
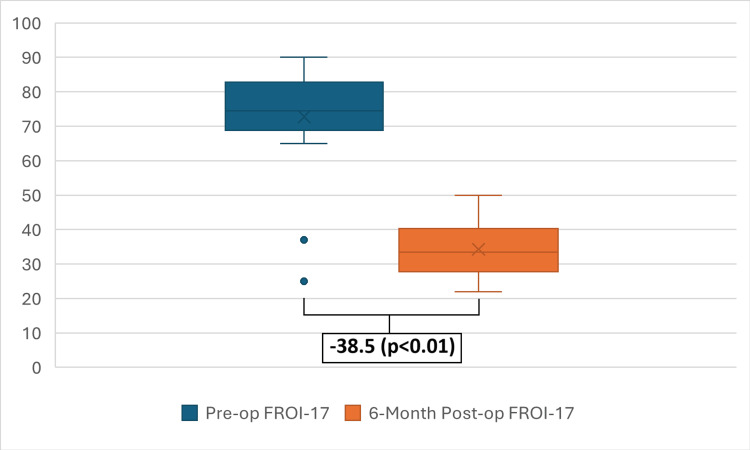
Difference Between Pre- and Post-procedure FROI-17 Scores FROI-17: Functional Rhinoplasty Outcome Inventory-17

**Figure 4:**
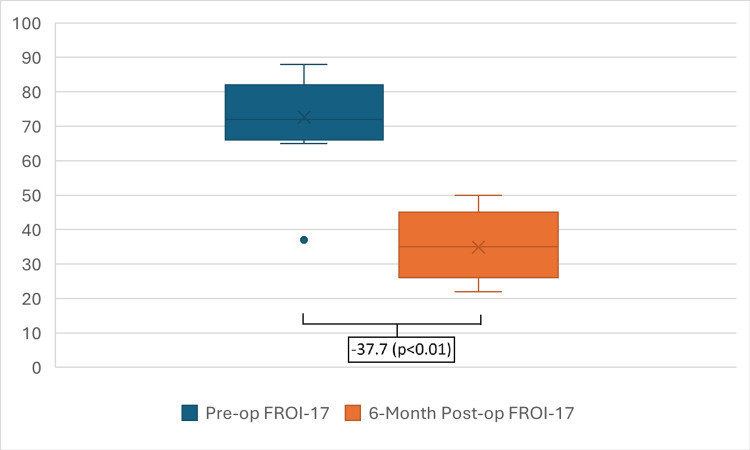
Difference Between Pre- and Post-procedure FROI-17 Scores for Patients Who Underwent Isolated LATERA Procedure FROI-17: Functional Rhinoplasty Outcome Inventory-17

A statistically significant improvement in mean FROI-17 score was observed, with a pre-operative mean of 72.8 (±15.5) decreasing to a post-operative mean of 34.3 (±7.9) at six months. This represents a mean decrease of -38.5 points (p < 0.01) (Figure 3). 90.9% of patients demonstrated a clinically significant improvement (>15-point decrement). This efficacy is consistent throughout different patient scenarios: GA versus LA and isolated LATERA procedures versus LATERA with concurrent procedures (Figure 4, Table 1). One patient reported no change in FROI-17 score, while another patient reported worsening of the score secondary to the development of a new septal deviation, requiring subsequent revision septoplasty.

**Table 1:** Effectiveness of LATERA Implant Across Different Scenarios FROI-17: Functional Rhinoplasty Outcome Inventory-17

Subgroup	Patients (n)	Mean ΔFROI-17	Key Insight
Local Anaesthesia	7	-36.7 points	Office-based treatment under local anaesthesia is highly effective, supporting its minimally invasive appeal.
General Anaesthesia	15	-39.3 points	Outcomes are excellent and no better or worse than local anaesthesia.
Isolated LATERA Procedure	11	-37.7 points	The implant alone provides substantial benefits for pure nasal valve collapse.
LATERA + Concurrent Surgery	11	-39.3 points	Works effectively as part of a combined functional surgical approach.

**Figure 5:**
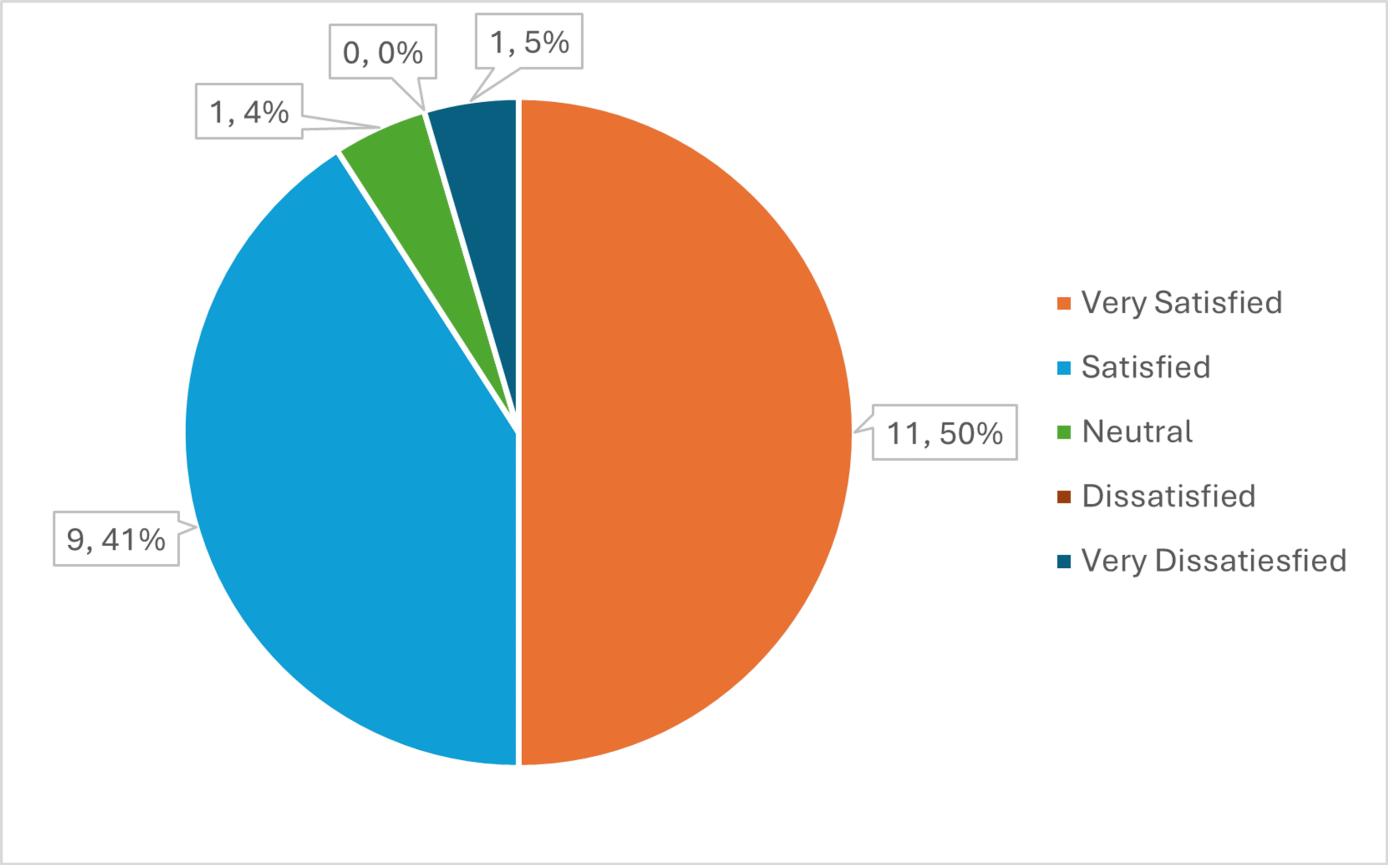
Distribution of Patients' Satisfaction at the Six-Month Follow-Up

At the six-month follow-up, 91% of patients reported being "Satisfied" or "Very Satisfied" with the procedure outcomes. The two patients with unchanged or worsened scores reported being "Dissatisfied" (Figure 5).

No major complications were reported; 4.5% experienced mild, transient inflammation at the insertion site, which resolved with conservative management within two weeks. There was no implant extrusion, infection, or persistent pain reported.

## Discussion

This prospective study demonstrates that the LATERA absorbable nasal implant is a safe and highly effective treatment for NVC in a district hospital setting. Our finding of a significant and clinically meaningful improvement in FROI-17 scores in 90.9% of patients aligns with the existing literature and provides real-world evidence for its efficacy [9-11]. The 38.5-point mean decrement overall and the 37.7-point mean decrement in the isolated LATERA procedure group exceed the 15-point MCID for the FROI-17 [7], underscoring the positive impact of the procedure on patients' quality of life.

When compared with the traditional surgical standard of cartilage grafting (e.g., spreader or alar batten grafts), the LATERA implant offers advantages that position it as an alternative in many clinical contexts, particularly for resource-conscious healthcare providers such as district hospitals.

LATERA achieves comparable efficacy with a good safety and recovery profile. Traditional grafting requires an invasive rhinoplasty approach, typically under general anaesthesia, and carries inherent risks. Revision rates for graft-related issues, such as displacement, warping, or resorption, are reported to be in the range of 5-10% [5]. In contrast, our data shows that LATERA, a minimally invasive procedure, achieves clinical improvement in over 90% of patients with no major complications (0% infection, extrusion, or persistent pain) and only transient minor inflammation in 4.5% of cases. This is consistent with multiple studies describing high procedural success and low complication rates [9-12]. This also translates to minimal downtime for the patient and a significant reduction in post-operative care burden.

The durability of the LATERA implant is supported by evidence from available literature. The implant is composed of poly-L-lactic acid (PLLA) and polycaprolactone (PCL), absorbable materials that provide a scaffold for the body to form a natural collagen matrix, ensuring permanent structural support after full absorption. The long-term efficacy and safety are illustrated by the 24-month randomised controlled trial (RCT) from Bikhazi et al., which demonstrated sustained improvement beyond 6 months without any implant-related failures [12]. This addresses a key question regarding the longevity of benefit from an absorbable device and confirms its role as a durable solution.

From a health economics and resource allocation perspective, LATERA offers transformative potential. Our subgroup analysis demonstrates that outcomes are comparable when the procedure is performed under local anaesthesia (-36.7 point improvement). This suitability for a true office-based application presents a paradigm shift. By moving the procedure out of the main operating theatre, LATERA reduces demand for theatre time, anaesthesia services, and recovery resources. It minimises patient absenteeism from work, facilitating a faster return to normal activities. These advantages directly align with strategic NHS priorities for expanding outpatient management, reducing elective surgery backlogs, and improving cost-effectiveness of care pathways [13,14].

LATERA provides a fundamentally different approach that decouples the treatment of NVC from the morbidity and resource intensity of formal rhinoplasty. For a district hospital like ours, where efficiency is paramount, it allows us to offer a highly effective, definitive procedure to a wider cohort of patients, including those unfit for or unwilling to undergo general anaesthesia, while optimising the use of limited hospital resources.

Limitations

We acknowledge the limitations of this study, including a small sample size, single-centre design, potential selection bias, and lack of a control group, which limit the generalisability of the findings. The six-month follow-up, while sufficient to demonstrate initial efficacy and safety, is inadequate to comment on the very long-term durability of the implant's effect, although the 24-month RCT data provide strong reassurance [12]. Future research should focus on larger, multi-centre and multi-ethnicity comparative studies with longer follow-up to directly compare LATERA with specific grafting techniques in a randomised fashion.

## Conclusions

The absorbable LATERA implant is a safe and highly effective treatment for NVC. In our study, we observed a statistically significant and clinically meaningful improvement in functional rhinoplasty-specific quality of life. Its minimally invasive application under local anaesthesia offers significant advantages over traditional cartilage grafting, including comparable efficacy, reduced revision rates, and minimal downtime. From a cost-effectiveness perspective, it reduces theatre and anaesthesia demands, aligning perfectly with NHS priorities for outpatient care and resource efficiency. LATERA represents a valuable tool in the functional surgeon's armamentarium and should be considered a compelling alternative option for suitable patients with NVC, particularly in resource-conscious healthcare settings.
